# Incidence and risk factors for surgical site infection following elective foot and ankle surgery: a retrospective study

**DOI:** 10.1186/s13018-020-01972-4

**Published:** 2020-10-01

**Authors:** Jinghong Meng, Yanbin Zhu, Yansen Li, Tao Sun, Fengqi Zhang, Shiji Qin, Haitao Zhao

**Affiliations:** 1 Department of Rheumatology and Immunology, The 3rd Hospital of Hebei Medical University, Shijiazhuang, 050051 Hebei People’s Republic of China; 2 Department of Orthopaedic Trauma Center, The 3rd Hospital of Hebei Medical University, Shijiazhuang, 050051 Hebei People’s Republic of China; 3 Department of Foot and Ankle Surgery, The 3rd Hospital of Hebei Medical University, No. 139 Ziqiang Road, Shijiazhuang, 050051 Hebei People’s Republic of China; 4 Department of Bone Tumor, The 3rd Hospital of Hebei Medical University, Shijiazhuang, 050051 Hebei People’s Republic of China

**Keywords:** Surgical site infection, Foot and ankle surgery, Multivariate analysis, Risk factors

## Abstract

**Background:**

This study aimed to investigate the incidence of surgical site infection (SSI) in elective foot and ankle surgeries and identify the associated risk factors.

**Methods:**

This was designed as a retrospective study, including patients who underwent elective surgery of foot and ankle between July 2015 and June 2018. Data on demographics, comorbidities, and perioperative parameters were collected from the medical records, the laboratory report, the operation report, and the outpatient follow-up registration database. SSI was defined in accordance with the Center for Disease Control criteria. Univariate and multivariate logistic regression analyses were used to identify the independent risk factors for SSI.

**Results:**

A total of 1201 patients undergoing 1259 elective foot/ankle surgeries were included, of whom 26 (2.1%) had an SSI, representing an incidence rate of 1.3% for superficial SSI and 0.8% for deep SSI, respectively. The results for organism culture showed *Pseudomonas aeruginosa* in 7 cases, *methicillin-resistant Staphylococcus aureus* (MRSA) in 6, *methicillin-susceptible Staphylococcus aureus* (MSSA) in 5, *methicillin-resistant coagulase-negative Staphylococci* (MRCNS) in 2, *Escherichia coli* in 2, and *Proteus mirabilis* in 1 case. Five factors were identified to be independently associated with SSI, including prolonged preoperative stay (OR, 1.21; 95% CI, 1.09 to 1.30), allograft or bone substitute (OR, 3.76; 95% CI, 1.51 to 5.30), elevated FBG level (OR, 1.17; 95% CI, 1.04 to 1.26), lower ALB level (OR, 2.33; 95% CI, 1.19 to 3.05), and abnormal NEUT count (OR, 1.72; 95% CI, 1.27 to 2.12).

**Conclusions:**

SSI following elective foot and ankle surgeries is low, but relatively high in forefoot surgeries, requiring particular attention in clinical practice. Although most not modifiable, these identified factors aid in risk assessment of SSI and accordingly stratifying patients and therefore should be kept in mind.

## Background

Surgical site infection (SSI) represents 31% of all hospital-acquired illness and is the most common nosocomial infection [1]. Specified in the elective orthopedic surgery, SSI was infrequent, with an incidence rate of 0.4 to 3.6% [2, 3]. But they can lead to serious consequence, such as bone union-related issues, joint dysfunction, amputation, and even increased mortality [4]. Besides, the substantial economic and social impact of infection-related complications should also be a concern. It was documented that, irrespective of surgery type, an infection could incur additional cost of £814 and £6626 due to prolonged hospitalization, wound care, or additional surgical debridement [5, 6]. Additionally, an infection was a major cause for readmission, accounting for 34.3% of the adverse affairs related to surgery [7].

Compared to other elective orthopedic surgeries (spinal procedure, total hip or knee arthroplasty), foot and ankle surgery had a relatively higher incidence rate of SSI, ranging from 0.5 to 6.5% [8]. Identification of risk factors associated with SSI could provide necessary information to appropriately counsel patients and aid surgeons to select targeted preventive measures. In the study by Ralte et al. [9], authors found the strict infection policy control policies that focused on surgical and environmental risk factors could reduce 50% of SSIs in elective foot and ankle surgery. By far, however, epidemiologic data on SSI following elective foot and ankle surgeries remain scarce. As far as we know, only very few studies focused on this subject and identified some risk factors, including alcohol use [10], preoperative narcotic use [10], advanced age [11], prolonged tourniquet time [11], tobacco use [11], complicated diabetes [4], perioperative antibiotics, and surgeon experience [12]. However, most of these risk factors were in controversy or inconclusive, which might be compromised by the small sample size or only inclusion of a specific population (diabetes mellitus, elderly, or foot and ankle trauma). It was possible that these variable results from studies might not be applicable to a general population undergoing elective foot and ankle surgeries.

Given that, we designed this study, with purposes to determine the incidence rate of SSI in patients undergoing clean elective foot and ankle surgery and secondly to identify the independent risk factors associated with SSIs.

## Materials and methods

### Study design, inclusion and exclusion criteria

This was a retrospective study and designed in accordance with the principles outlined in the Declaration of Helsinki. It had been approved by the institutional review board of the 3rd Hospital of Hebei Medical University. Between July 2015 and June 2018, all patients aged 18 years or older who underwent clean elective foot and ankle surgery were confirmed by querying electronic medical records (EMR). The exclusion criteria were as follows: foot and ankle trauma, revision surgery, isolated ankle arthroscopy, hardware removal, previous foot/ankle surgery, foot/ankle ulcerations, patients with incomplete EMR data, death within the hospitalized stay, or follow-up period < 12 months.

According to our policy, patients who are to be operated are compulsory to soak their feet in the simple soap solution the night before the operation, but are no need of hair removal. Then, before surgery, the surgical site is cleansed with an antiseptic soap solution and broad disinfection with iodine solution is performed by three times. Generally, single dose of prophylactic antibiotic (cefuroxime, ceftriaxone, or ceftazidime) is administered intravenously 30 min before the surgery, and in some cases exceeding 3 h, a second dose is given.

### Definition of SSI and confirmation of cases

SSI is confirmed by the documented signs or symptoms of infection in the EMR and bacteria culture results or drug sensitivity available in the microbial culture report. SSI is diagnosed based on the criteria proposed by the Center for Disease Control (CDC) [13], wherein SSI is an incisional infection that occurs within 30 days after surgery if no implant is left in place or within 1 year if an implant is left in place. SSI is classified as deep and superficial SSI. The deep SSI involves deep soft tissue and meets at least one of the following: persistent wound discharge or dehiscence; visible abscess or gangrenosis that requires surgical debridement, implant exchange, or removal; and an infection that involves only the skin or subcutaneous tissue, presenting wound problem (redness, swelling, hot, pain) but does not meet the diagnosis criteria of deep SSI is deemed to be a superficial SSI.

We confirm the SSI cases if we found they were (1) documented by the treating surgeon in the inpatient medical record, (2) documented in the outpatient follow-up registration database, and (3) confirmed by the telephone visit by the end of 1 year postoperatively. It is of note that SSI cases which were treated in our institution and in other institutions or untreated to resolve were all included in this study.

### Data collection

Two researchers (Y Li and T Sun) inquired patients’ EMR, scheduled follow-up registration database and microbial culture reports for patients’ demographics, lifestyles, comorbidities, surgery-related data, and laboratory biomarkers.

The detailed data for each patient were age, sex, body mass index (BMI), tobacco use, alcohol consumption, and comorbidities (hypertension, diabetes mellitus (DM), chronic heart disease, chronic pulmonary disease, peripheral vascular disease, and rheumatoid disease). Due to the retrospective design, pack-year history of tobacco users or volume of alcohol consumption was not available. BMI (kg/m^2^) was grouped based on the criteria suitable for Chinese populations: < 18.5 (underweight), 18.5–23.9 (normal), 24.0–27.9 (overweight), and ≥ 28.0 (obesity) [14, 15]. Comorbidities were confirmed mainly based on the patients’ self-reported disease history at their admission. Also, the repeated measurements of blood pressure, fasting blood glucose (FBG), glucose tolerance tests, or glycosylated hemoglobin levels were used for additional diagnosis of hypertension or DM.

Surgery-related variables included preoperative stay (between admission and operation), surgeon level (senior, vice senior, attending surgeon, or resident), American Society of Anesthesiologists classification (ASA), anesthesia type, surgical duration, use of allogeneic bone or bone substitute, volume of intraoperative blood loss, intraoperative blood transfusion, intraoperative or postoperative prophylactic use of antibiotics, and postoperative drainage.

The following laboratory biomarkers were collected: preoperative white blood cell (WBC), neutrophil (NEUT), lymphocyte (LYM), red blood cell (RBC), hemoglobin (HGB), hematocrit (HCT), total protein (TP), albumin (ALB), globulin (GLOB), A/G value, and FBG. If patients have multiple laboratory biomarkers recorded during the preoperative period, the biomarkers measured at closest date before surgery were selected.

### Statistical analysis

The continuous data were expressed as mean ± standard deviation (SD) or median (interquartile range, IQR) and were evaluated consistently by the Student *t* test or Mann-Whitney *U* test, when appropriate. The categorical data were expressed as number and percentages and were analyzed by chi-square or Fisher’s exact test, when appropriate.

The variables that were tested to be significant at statistical level *p* < 0.1 in the univariate analyses were further entered into a multivariate logistic regression model to determine their independent effect on SSI, using stepwise backward elimination method. The goodness-of-fit of the final model was evaluated by the Hosmer-Lemeshow test, with a *p* > 0.05 indicating an acceptable result, and was quantitatively evaluated by Nagelkerke’s *R*^2^, with greater value indicating a better result. SPSS 23.0 software package (SPSS Inc., Chicago, IL) was used to analyze all the data.

## Results

### General result

Totally, 1201 patients who underwent 1259 elective surgeries at foot/ankle were included. Of them, there were 572 males (47.6%) and 629 females (52.4%). The mean age was 45.0 years (SD, 16.2), with patients of 30 to 64 years in predominance (948, 79%). The most commonly performed procedure was osteotomy (497, 45.8%), followed by arthrodesis (392, 31.1%), soft tissue procedure (63, 5.0%), and combined procedure (307, 18.0%). Surgical procedure was performed at median of 2 days (IQR, 1 to 4 days) after admission, and 83.0% (1045/1259) were performed within 3 days after admission. The median total hospital stay was 8.0 days (IQR, 6 to 13 days).

### Characteristics of SSI (incidence, causative bacteria type, and SSI occurrence time)

Twenty-six SSIs occurred in 26 patients, representing an overall incidence rate of 2.1%. Of them, 16 (incidence rate, 1.3%) were superficial and 10 (incidence rate, 0.8%) were deep SSI. The positive results of organism culture for 10 deep and 14 superficial SSIs showed *Pseudomonas aeruginosa* in 7, *methicillin-resistant Staphylococcus aureus* (*MRSA*) in 6, *methicillin-susceptible Staphylococcus aureus* (*MSSA*) in 5, *methicillin-resistant coagulase-negative Staphylococci* (*MRCNS*) in 3, *Escherichia coli* in 2, and *Proteus mirabilis* in 1 case. There were no SSIs that were caused by mixed bacteria. The median time at which SSIs occurred was 5.5 days after operation, ranging from 2 to 38 days postoperatively.

Stratified by surgical location, most SSIs occurred in the forefoot (22/26, 84.6%) and 21 were cultured positive for causative organisms, with *Pseudomonas aeruginosa* in 6 cases, *MRSA* in 6, *MSSA* in 4, *MRCNS* in 2, *Escherichia coli* in 2, and *Proteus mirabilis* in 1 case; one SSI caused by *MSSA* occurred at the midfoot; 2 among 3 SSIs in the hindfoot were cultured positive, with one caused by *MRCNS* and the other by *Pseudomonas aeruginosa*.

### Univariate analyses

The results of univariate analyses are presented in Table 1. There were significant differences between the SSI and non-SSI groups in terms of age (*p* = 0.040), diabetes mellitus (*p* < 0.001), preoperative stay (*p* < 0.001), intraoperative bleeding (*p* = 0.006), anesthesia type (*p* < 0.001), type of bone graft (*p* = 0.002), albumin (*p* < 0.001), A/G (*p* < 0.001), ALT (*p* = 0.019), AST (*p* = 0.011), ALP (*p* = 0.020), GLOB (*p* = 0.002), FBG (*p* = 0.042), WBC (*p* < 0.001), NEUT (*p* < 0.001), RBC (*p* < 0.001), HGB (*p* < 0.001), MCHC (*p* < 0.001), PLT (*p* < 0.001), and PDW (*p* < 0.034). The total hospital stay was 26.2 days in patients with SSI, while 10.3 days in those without SSIs (*p* < 0.001).

**Table 1:** Association between potential risk factors and surgical site infection (SSI) in elective foot and ankle surgery

Variables	Number (%) of infection	Number (%) of non-infection	***p*** value
**Gender (male)**	13 (50.0)	576 (46.7)	0.740
**Age (years)**	40.3 ± 11.5	45.3 ± 16.4	0.040
**Living place**			0.311
Rural	16 (61.5)	635 (51.5)	
Urban	10 (38.5)	598 (48.5)	
**BMI (kg/m**^**2**^**)**	24.4 ± 5.6	25.4 ± 3.9	0.280
18.5–23.9	8 (30.8)	437 (35. 5)	0.112
< 18.5	3 (11.5)	38 (3.1)	
24.0–27.9	7 (26.9)	472 (38.3)	
28.0–31.9	6 (23.1)	232 (18.8)	
≥ 32.0	2 (7.7)	54 (4.4)	
**Diabetes mellitus**	7 (26.9)	96 (7.8)	< 0.001
**Hypertension**	4 (15.4)	259 (21.0)	0.621
**Chronic heart disease**	3 (11.5)	62 (5.0)	0.138
**Allergy history**	2 (7.7)	132 (10.7)	0.622
**Preoperative stay (days)**	5.4 ± 3.4	2.8 ± 2.9	< 0.001
**Total hospital stay (days)**	26.2 ± 10.4	10.3 ± 11.9	< 0.001
**Intraoperative bleeding (mL)**	45.6 ± 83.2	71.8 ± 140.1	0.006
**Surgical duration (min)**	76.5 ± 47.0	64.8 ± 51.4	0.074
**Cigarette smoking**	8 (30.8)	218 (17.7)	0.085
**Alcohol consumption**	5 (19.2)	402 (32.6)	0.149
**Surgeon level**			0.403
Attending or resident	5 (19.2)	167 (13.5)	
Senior or vice senior	21 (80.8)	1066 (86.5)	
**Surgical location**			0.233
Forefoot	22 (84.6)	869 (69.0)	
Midfoot	1 (3.8)	97 (7.7)	
Hindfoot	3 (11.5)	293 (23.3)	
**Procedure**			0.677
Osteotomy	9 (34.6)	488 (39.6)	
Arthrodesis	7 (26.9)	385 (31.2)	
Soft tissue	1 (3.8)	62 (5.0)	
Combined two or three above	9 (34.6)	298 (24.2)	
**Anesthesia**			< 0.001
Local	6 (23.1)	585 (47.4)	
Spinal	12 (46.2)	564 (45.7)	
General	8 (30.8)	84 (6.8)	
**Type of bone graft**			0.002
None	21 (80.8)	1150 (93.3)	
Autograft	3 (11.5)	68 (5.5)	
Allograft or bone substitute	2 (7.7)	15 (1.2)	
**ASA class**			0.123
I	6 (23.1)	520 (42.2)	
II	18 (69.2)	680 (55.2)	
III or greater	2 (7.7)	33 (2.7)	
**Intraoperative antibiotics**	16 (61.5)	791 (64.2)	0.779
**Postoperative antibiotics**	17 (65.4)	724 (58.7)	0.494
**Drainage use**	19 (73.1)	713 (57.8)	0.119
**TP (< 60 g/L)**	2 (7.7)	65 (5.3)	0.229
**ALB (< 35 g/L)**	7 (26.9)	18 (1.5)	< 0.001
**GLOB (> 40 g/L)**	4 (15.4)	43 (3.5)	0.002
**A/G (< 1.2)**	7 (26.9)	64 (5.2)	< 0.001
**ALT (> 40 U/L)**	4 (15.6)	62 (5.0)	0.019
**AST (> 35 U/L)**	4 (15.4)	47 (3.8)	0.011
**ALP (> upper limit)**	2 (7.7)	17 (1.4)	0.020
**FBG (mmol/L)**	5.9 ± 2.8	5.3 ± 1.4	0.042
**WBC (> 10 × 10**^**9**^**/L)**	3 (11.5)	44 (3.6)	< 0.001
**NEUT (> 6.3 × 10**^**9**^**/L)**	5 (19.2)	59 (4.8)	< 0.001
**LYM (> 3.2 × 10**^**9**^**/L)**	3 (11.5)	86 (7.0)	0.468
**RBC (< lower limit)**	12 (46.2)	14 (53.8)	< 0.001
**HGB (< lower limit)**	16 (61.5)	10 (38.5)	< 0.001
**HCT (< lower limit)**	4 (15.4)	97 (7.9)	0.163
**MCHC (< lower limit)**	4 (15.4)	22 (84.6)	< 0.001
**PLT (< lower limit)**	2 (7.7)	24 (92.3)	< 0.001
**PDW (< lower limit)**	3 (11.5)	23 (88.5)	0.034

*RBC* red blood cell, reference range: females 3.5–5.0/10^12^/L and males 4.0–5.5/10^12^/L; *HGB* hemoglobin, reference range: females 110–150 g/L and males 120–160 g/L; *ALB* albumin; *FBG* fasting blood glucose; *ALP* alkaline phosphatase, 50–135 u/L; *HCT* hematocrit, 40–50%; *MCHC*, mean corpuscular hemoglobin concentration, 316–354 g/L; *WBC* white blood cell; *NEUT* neutrophil; *LYM* lymphocyte; *PLT* platelet, 100–300 × 10^9^/L; *TP* total protein; *ALB* albumin; *GLOB* globulin; *PDW* platelet distribution width, reference 12–18.1%

### Multivariate analyses

We entered significant variables together with surgical duration (*p* = 0.074) and cigarette smoking (*p* = 0.085) into the multivariate logistic regression model. After adjustment for confounding factors, five independent risk factors were identified to be associated with SSI. Delay of each day before operation was associated with 21% increased risk of SSI (OR, 1.21; 95% CI, 1.09 to1.30). With reference to normal FBG level (< 6.1 mmol/L), the risk of SSI was increased by 17% with each increment of 1 mmol/L (OR, 1.17; 95% CI, 1.04 to1.28). Compared to normal ALB level (≥ 35 g/L) or NEUT count (1.8–6.3 × 10^9^/L), the abnormal level was associated with 2.33 times and 1.72 times increased risk of SSI, respectively. The risk of SSI in patients with implantation of allograft or bone substitute was 3.76 times as those with none (OR, 3.76; 95% CI, 1.51–5.30). The results are detailed in Table 2. The Hosmer-Lemeshow test showed the adequate fitness of the final model (*χ*^2^ = 4.469, *p* = 0.527; Nagelkerke’s *R*^2^ = 0.377).

**Table 2:** Multivariate analyses of risk factors associated with SSI in elective foot and ankle surgery

Variable	OR (95% CI)	***p*** value
Preoperative stay (increase of each day)	1.21 (1.09 to 1.30)	0.002
Allograft or bone substitute	3.76 (1.51 to 5.30)	< 0.001
ALB level < 35 g/L	2.33 (1.19 to 3.05)	0.021
NEUT > 6.3 × 10^9^/L	1.72 (1.27 to 2.12)	0.039
FBG level (increase in each unit)	1.17 (1.04 to 1.26)	< 0.001

*ALB* albumin, *NEUT* neutrophil, *OR* odds ratio, *CI* confidence interval

## Discussion

In this study, we found the overall incidence rate of SSI following elective foot and ankle surgery was 2.1% (1.3% for superficial and 0.8% for deep SSI). Several independent risk factors associated with SSI were identified, including prolonged preoperative stay, implantation of allograft or bone substitute, elevated FBG level, lower ALB level (< 35 g/L), and increased NEUT count. Patients with SSI had 2.5 times prolonged total hospital stay as those with non-SSI (26.2 vs 10.3 days).

The incidence rate of SSI in foot and ankle surgery was reported to be varied, from 1.4 to 13.2% [4, 8, 9], largely due to the differences in definitions of SSI, study design, medical conditions, interventions, or follow-up period. Wiewiorski et al. [11] conducted a prospective study of 290 elective foot and ankle procedures and found the overall prevalence of wound complication of 16.9%, with 1.4% for deep infections requiring irrigation and debridement. Zgonis et al. [8] evaluated the efficacy of prophylactic use of antibiotics in elective foot and ankle surgery, but did not find the significant difference of SSI rate between patients who received preoperative antibiotic use and those did not (1.6% vs 1.4%). In another study focusing on DM patients, SSI rate of 3.5% was observed, being 9.5% in DM patients and 2.4% in non-DM patients (*p* < 0.001) [16]. Despite with different settings, these studies provided more data to counsel patients about risk of SSI, facilitating the operative decision-making and perioperative optimal interventions.

Compared to the elective knee and hip arthroplasty, the incidence rate of SSI following elective foot and ankle surgery was higher, approximately 5 to 10 times high as that [3, 17]. Ralte et al. [9] attributed this to the abundance of eccrine sweat glands and the hot and humid environment that caused microbiological flora in the feet. Additionally, the anatomical relationship of the toes is also an important contributor, which might weaken the efficacy of the preoperative skin disinfection. Tachibana [18] found the forefoot was more densely populated with microorganisms than other areas of the foot. Furthermore, it had been demonstrated that eliminating bacteria from the forefoot prior to surgery was more difficult [19, 20]. In this study, we found a trend towards a higher incidence of SSI following forefoot surgeries (2.5% vs 1.0%), although the result was non-significant, which, we thought, was primarily decided by the limited number of SSI cases (*n* = 26). Despite this, the future attention should focus on optimization of local surgical conditions and formation of a more reasonable antibiotic prophylaxis program aimed at the forefoot.

In the present study, higher FBG level rather than diagnosis of previous DM was identified as an independent risk factor for postoperative SSI, and increase of 1 mmol/L in FBG was associated with 17% increased risk of SSI. This seemingly contradictory result did reflect the importance of blood glucose control for the prevention of SSI in patients with DM. Indeed, the role of history of DM in SSI has always been in controversy. In the study by Wiewiorski et al. [11], authors did not observe the independent association of SSI with DM in elective foot and ankle surgeries, which should be treated in the context of inadequate samples (*n* = 290). In a prospective cohort of 1465 foot and ankle surgeries conducted by Wukich et al. [16], DM-related peripheral neuropathy rather than DM itself was identified to be strongly associated with the development of SSI. Similarly, in a study of 21,854 diabetic patients, the authors found the number of comorbid conditions associated with DM had more predictive value than either DM or blood glucose level for occurrence of SSI [21]. These findings suggested that strict glycemic control (or HbA1c) and identification of comorbid conditions associated with DM made more sense than DM diagnosed by medical history, and they could become a routine part of preoperative comorbidity management.

The relationship between hypoalbuminemia and postoperative complications has been well established in orthopedic surgery and other subspecialties of surgery [22, 23], but was firstly reported as far as we know. Generally, patients who underwent elective foot and ankle surgeries were younger than those undergoing hip or knee arthroplasty, and in a certain proportion of hospitals, albumin was not routinely tested. In this study, the average age of included patients was 45 years, and only 2% of them had hypoalbuminemia (25/1259) in the context of routine test of preoperative albumin. Therefore, in such a low proportion (2%), it might be very difficult to obtain the significant result via multivariate analyses in studies with small sample or without readily available albumin data. By far, several studies have demonstrated the improved outcomes via optimization of nutritional status prior to major surgery, including abdominal surgery, arthroplasties, or orthopedic trauma [24–26]. However, serum albumin is more often used to be a prognostic or nutritional marker, because simply restoring albumin level might not improve clinical outcomes [27].

Allograft or bone substitute as a risk factor for SSI was also firstly reported in this study and had the strongest association (OR, 3.76). On the one hand, the increased tension of skins around operative site or operative cavity after implantation of allograft or bone substitute may cause effusion of tissue fluid [28]. On the other hand, the graft of tissues does not have blood flow [29], which should be also considered as a potential cause for infection. But due to the limited cases (17 cases), this association was not definitive and its mechanism requires to be further investigated. The role of preoperative stay in risk of SSI in elective foot and ankle surgery should be rationally treated because the causes might be multifactorial, such as more time needed for optimization of comorbidities, unavailability of operation resources, weekends, or holidays [30, 31]. About 5% of patients in this study had an increased neutrophil count (> 6.3 × 10^9^/L), in whom the incidence rate of SSI was 7.8%, significantly higher than those with normal range of neutrophil count. In other studies of elective arthroplasties or others, the similar results were found [32, 33]. These 3 factors might not be modified or optimized pertinently, but they could provide more references for surgeons to evaluate the risk of SSI following elective foot and ankle surgeries.

The major strengths of this study included its large sample size and multiple factors included for adjustment. Some limitations should also be mentioned. Firstly, the retrospective design compromised the accuracy and reliability in data collection, because the data depended on the documentations in the EMR. Secondly, the incidence rate of SSI might be underestimated due to our imperfect follow-up strategy. It was likely that one patient who developed a slight SSI that was untreated to resolve might report no SSIs occurring. Thirdly, as with every multivariate logistic regression analysis, there remain residual confounding effects, because of the unmeasured or unanticipated variables. Fourth, we could not obtain the data on patients’ surgical site care or compliance with medication and functional exercises.

In summary, the incidence rate of SSI following elective foot and ankle surgery was low, but relatively high for forefoot surgeries. Prolonged preoperative stay, allograft or bone substitute, elevated FBG level, ALB level, and NEUT count were identified to be independently associated with SSI. Although not modifiable, they do help in patient counseling, SSI risk assessment, and stratifying patients, and should be kept in mind through perioperative period.

## Data Availability

All the data will be available upon motivated request to the corresponding author of the present paper.

## Abbreviations

SSI: Surgical site infection
BMI: Body mass index
EMR: Electronic medical records
ASA: American Society of Anesthesiologists
CDC: Center for Disease Control
DM: Diabetes mellitus
SD: Standard deviation
OR: Odds ratio
RBC: Red blood cell
HGB: Hemoglobin
ALB: Albumin
FBG: Fasting blood glucose
ALP: Alkaline phosphatase
HCT: Hematocrit
MCHC: Mean corpuscular hemoglobin concentration
WBC: White blood cell
NEUT: Neutrophil
LYM: Lymphocyte
PLT: Platelet
TP: Total protein
GLOB: Globulin
PDW: Platelet distribution width
MRSA: *Methicillin-resistant Staphylococcus aureus*
MSSA: *Methicillin-susceptible Staphylococcus aureus*
MRCNS: *Methicillin-resistant coagulase-negative Staphylococci*
